# Supplementary material for: Are carbon emissions trading and green financial instruments synergistic? -Comprehensive quantitative research based on content analysis
Source: PLoS One. 2024 Mar 7;19(3):e0298601. doi: 10.1371/journal.pone.0298601 (PMC10919601; doi:10.1371/journal.pone.0298601)
Supplement: S1 Data — (ZIP) [file pone.0298601.s001.zip › Data/地级市Z_F工作报告.zip!/┴╔─■/╕╖╨┬╩╨/└┤╫╘╣1⁄2╓┌║┼í╛┬φ┐╦╩2╛▌═°í┐_2011.docx]

各位代表：

　　现在，我代表市政府向大会报告工作，请予审议，并请市政协各位委员和其他列席同志提出意见。

　　一、“ 十一五”时期及2010 年政府工作回顾

　　“十一五”时期，在市委的正确领导下，全市各族人民团结一心，奋力拼搏，全面实施“突破阜新”战略，深入推进转型振兴进程，胜利完成了“十一五”规划的目标任务。

　　过去的五年，是经济综合实力持续增强的五年。地区生产总值年均增长15.3%，地方财政一般预算收入年均增长25.7%，全社会固定资产投资年均增长36.7%。主要指标实现翻番，全市经济进入了快速发展的新时期。

　　过去的五年，是产业结构调整成效明显的五年。工业经济比重提高7.1 个百分点，装备制造、农产品加工等接续替代产业初步形成。传统服务业改造提升，现代服务业快速发展。农业产业化步伐加快，社会主义新农村建设扎实推进。三次产业结构呈现二三一格局，经济转型试点工作取得重要成果。

　　过去的五年，是城乡基础设施显著改善的五年。沈彰等3条高速公路建成通车，总里程跃居全省前列。城乡公路网络不断完善，引白水源等一批重点工程投入使用，城市供水、供热、供气保障水平不断提高。城乡面貌发生重大变化，新城建设全面启动，县城和小城镇建设成效明显。森林覆盖率达到32.1% ，城市绿化覆盖率达到40.5%。完成国家下达的节能减排目标。环境空气质量达到国家二级标准天数由2005年的237天增加到2010年的337天。

　　过去的五年，是改革开放不断深入的五年。对外开放环境日趋优化，累计招商引资到位额383.5 亿元，直接利用外资2.7亿美元，出口总额4.6亿美元，分别是“十五”时期的2.9倍、2.3倍和3.7倍。加入沈阳经济区，为我市在对外开放与合作上搭建了广阔平台。192户国有企业实现转制，财政金融、行政管理等领域改革取得新进展，集体林权制度主体改革基本完成。

　　过去的五年，是人民群众得到更多实惠的五年。累计实现城镇实名制就业33.4万人次。社会保障覆盖面进一步扩大，城乡救助体系不断完善。改造棚户区377 万平方米，援建和维修农村贫困户住房6000余栋。城镇居民家庭人均可支配收入、农村居民家庭人均纯收入分别是2005 年的1.9 倍和2.1 倍。

　　各项社会事业协调发展，民主法制建设得到加强，社会和谐稳定。

　　各位代表，刚刚过去的一年，是“十一五”期间发展成果最为集中体现的一年。预计实现地区生产总值355 亿元，增长16.5%；地方财政一般预算收入30.1亿元，增长29.8%；全社会固定资产投资350亿元，增长45.8%；社会消费品零售总额148.9 亿元，增长18.6%；直接利用外资1.1 亿美元，增长72%；出口总额1.2 亿美元，增长13%；城镇居民家庭人均可支配收入12690 元，增长13.5%；农村居民家庭人均纯收入6500 元，增长20.8%。主要经济指标增幅高于全省平均水平。

　　（一）“三大基地”和“六个重点产业集群”建设实现新突破

　　全市期盼的大唐煤制气一期项目全面开工，基地建设呈现设备林立、机声轰鸣的壮观景象。巴新铁路累计投资25 亿元。风电装机容量突破百万千瓦，新疆金风、韩国尤尼深等风电设备制造项目开工，华能等3 个兆瓦级光伏发电示范项目启动。山东鲁花、江苏雨润、内蒙伊利等知名品牌项目齐聚阜新。

　　“六个重点产业集群”新开工项目290个，竣工投产项目206个，实现产值177 亿元。其中，液压产业集群产值突破百亿元，铸造产业集群产值达到40亿元。

　　企业“倍增计划”深入实施，阜矿集团打造百亿元企业目标成功实现，总收入达到128 亿元，增长63.9%。阜新发电公司一举扭转连续五年亏损局面。供电公司电力保障能力不断增强。力昌钢铁等71 户企业主营业务收入实现当年翻番。循环经济加快发展，天合环保粉煤灰综合利用等10 个项目开工建设，再生资源循环经济产业基地启动。规模以上工业企业净增105 户，达到500 户；增加值突破百亿元，达到115 亿元，增长18.5%。民营经济增加值、税金分别增长27.1%和25.4%。国税、地税收入均突破20亿元。

　　“ 千个项目大会战”任务全面完成，实施投资千万元以上项目1006个。

　　（二）现代农业和县域经济快速发展

　　粮食产量46.4 亿斤，创历史最好水平。花生种植面积达到217万亩，增长50.2%。新建保护地5 万亩，新增畜禽标准化小区285 个。

　　生猪、奶牛、肉牛、肉鸡等重点基地供应能力增强，南瓜、树莓、酿酒葡萄等特色基地规模和产业化水平明显提升。

　　人工造林和封山育林95万亩，清理“小开荒”16.5 万亩，章古台自然保护区晋升为国家级自然保护区。国有林场改革工作取得初步成效。

　　建设各类小型水源3848处，新增灌溉面积4.9 万亩。完成5 万亩河道生态建设和23 座病险水库除险加固工程，阜彰两县被确定为国家小型农田水利重点县。乡镇气象站建设得到加强，人工增雨能力进一步提高。农田作业综合机械化水平达到72%。

　　县域经济“三年倍增计划”圆满完成，阜彰两县财政一般预算收入分别突破4亿元和3亿元。

　　（三）服务业和城区经济发展迈出新步伐

　　加快推进以中央商务区为重点的服务业聚集区建设，兴隆百货二期全面启动，东市地下商业街、解放大街地下商业城主体完工，大商百货大楼正式营业。开发区汽车商贸聚集区初具规模，新邱商业步行街投入运营，站前现代城开工。投资100亿元的葫芦岛港物流产业园区项目落户。“早餐工程”、“万村千乡”市场工程、“家电下乡”、“家电以旧换新”等惠民工程积极推进。

　　房地产施工面积400 万平方米，完成投资35 亿元，增长71.6%。海州露天国家矿山公园被命名为国家科普基地，孙家湾国际工业遗产旅游度假区开工，纵横滑雪场等项目投入运营。“温泉旅游新城”建设进展顺利，天水谷温泉旅游度假村等7个项目落户。瑞应寺景区环寺路等3 个项目投入使用。各金融机构新增贷款60亿元，为全市经济发展作出重要贡献。

　　阜新银行沈阳分行开业。新建阜蒙县农商村镇银行。新成立小额贷款公司20 家、融资性担保机构6 家，激活民间资本16.6亿元。大金重工成功上市，实现我市企业境内上市零的突破。

　　五个城区生产总值平均增幅高于全市5.5个百分点，财政一般预算收入均超过亿元。

　　（四）对外开放水平进一步提高

　　组织赴北欧、香港等8次“走出去”活动，举办“千户企业阜新行”活动周、“玉龙新城”招商推介会等5 次“请进来”活动，借助省直部门帮扶平台，签约项目227 个，中航、绿地等国际、国内500 强企业成功落户。招商引资到位额150 亿元，增长70%。新能源产业继续成为外资投向的热点，占外资到位额的62%。外贸出口超百万美元企业新增3户，达到10户。开发区、高新区对外开放先导区作用进一步增强。

　　（五）城市建设力度不断加大

　　全面实施城市发展战略规划，“五个新城”等43个专业、专项规划编制完成，规划的龙头作用明显增强。

　　“玉龙新城”基础设施建设全面展开，施工面积达到98 万平方米。

　　“沈彰新城”滨河路等主干路开工建设。建筑业产值达到85亿元，增长45%。

　　京沈客专、锦阜高铁路扩能改造项目开工在即，阜盘、彰通高速公路路基和桥梁工程完工，沈彰开发大道、皮革基地快速通道开工建设，沈彰通客专、军民合用机场等项目前期工作进展顺利。对工业街等26 条街巷路进行维修改造，五龙公铁立交桥、惠宾大桥通车。

　　加快推进环城水系建设，细河景观得到较大改善，玉龙湖开发和九营子河、高林台河治理工程开工。新增城市绿化面积174万平方米，创历史新高。更新公交车辆100 台，新增、改造公交线路9条。实施供水等地下管网改造110公里，“三环制”供热改造全面完成。污水再生回用工程及县区3个污水处理厂投入使用，垃圾处理厂二期工程开工。积极开展国家卫生城创建工作，市容环境综合整治夏季行动成效明显，城市面貌不断改善。

　　（六）民生工程稳步推进

　　实现城镇实名制就业5.8 万人，城乡劳动力转移31.2万人次，创建省级创业型城市工作全面启动。

　　社会保险覆盖面和保障水平进一步提高，彰武县新型农村社会养老保险试点参保人数达到17.1万，企业退休人员基本养老金月人均提高123 元。新农合覆盖率达到97%。

　　城、乡低保对象救助标准分别提高20%和35%，农村五保对象救助标准年人均提高360元。棚户区改造开工面积83万平方米，建设廉租住房、经济适用住房5080套。归集住房公积金突破20 亿元。加强价格监管，保障市场供给，物价水平基本稳定。

　　建设市民健康广场50个，城区20 所中小学新建塑胶操场投入使用，60 个老旧小区综合整治、63 万平方米居民楼供热计量和节能改造完成。新建公厕21 座、农村改厕2 万座，城区中小学改厕全部完成。

　　社区“十分钟服务圈”实现全覆盖，农村示范社区达到100 个。设立“阜新慈善日”，成立慈善物资调剂中心和7 家慈善超市。市新殡仪馆、流浪儿童救助保护中心综合楼投入使用。改造区域性中心敬老院5所，完善100个社区夕阳红老年图书室。新建农村乡镇综合文化站13 个、农家书屋318个、甲级村卫生室300所。减少农村低收入人口10万人。新建一村一眼“保命井”50眼，解决8.6万农村人口饮水安全问题。

　　（七）社会事业协调发展

　　新增省级以上研发中心3 个、企业技术中心4 个，开发高新技术产品100 项，高新技术产品增加值达到12亿元，增长50%。液压创业服务中心被科技部认定为国家高新技术创业服务中心。

　　新邱区通过省新一轮“双高”普九验收。新建农村九年制学校22 所，改造维修校舍30.4 万平方米。

　　市蒙中小学部建成。教育资源整合力度加大，辽工大北校区投入使用，阜新高专通过置换办学条件得到较大改善，市职业教育集团成立。

　　现代评剧《东藏圣火》获全国第七届评剧节银奖，大型音乐舞蹈诗《悠悠玉龙情》获省第八届艺术节金奖，重大历史题材影片《辛亥革命》在阜开机拍摄，成功举办第五届玛瑙博览会，市第三届职工文化技术节和“市长杯”职工技能大赛促进了广大职工综合素质提高，《我爱红诗》走进阜新活动激发了市民热爱家乡的热情。彰武县被中华诗词学会誉为全省唯一的“ 中国诗词之乡”。整合后的市第二人民医院运营良好，阜矿总医院外科大楼投入使用，市体育馆建成并向市民开放，市工人文化宫改造完成，市中心医院新病房楼、人口和计划生育服务中心楼、图书馆等工程主体完工。

　　精神文明建设深入开展，市民文明程度不断提高。民族宗教工作得到加强。双拥共建工作取得新成效。第六次全国人口普查进展顺利。强化社会治安综合治理，依法严厉打击各类违法犯罪活动，人民群众安全感不断增强。信访包案责任制深入实施，各类社会矛盾有效化解。安全生产形势总体平稳，地方煤矿实现460 天无死亡事故，创历史最好水平。

　　工商、烟草、质监、邮政、通信、石油等中省直单位为地方经济发展作出积极贡献，外事侨务、民防、民兵预备役、地震、地方志、档案、老龄、残疾人、妇女儿童、红十字会等工作取得新成绩。

　　（八）政府自身建设进一步加强

　　自觉接受人大依法监督和政协民主监督，坚持重大事项向人大报告、政协通报制度，人大代表议案、建议和政协提案办复率、满意率均达100%。认真听取各民主党派、工商联、无党派人士和人民团体的意见。

　　行政效能监察和行政权力运行制度建设取得明显成效，审计监督力度加大，国有资产监管工作不断规范。“网络直评部门”活动启动，绩效考评激励作用增强。政府机构改革深入开展，事业单位岗位管理制度改革全面推行。市以下财政管理体制进一步完善，县区在地统计全面实施。第十届村委会换届选举工作顺利完成。

　　推行全程代理制，行政审批效率明显提高。对政务公开、电子政务、信息发布、行政诉讼职能进行整合，电子政务网络平台应用水平进一步提高。在中国阜新门户网站开通政府论坛，通过市长公开电话、市长信箱、市长联络员等渠道，解决群众诉求2.3万件。

　　各位代表，“十一五”以来，我们经历了复杂多变的形势，走过了艰苦创业的历程，创造了令人瞩目的成绩。五年取得的成绩来之不易，积累的经验弥足珍贵。这些成绩的取得，是在省委、省政府和市委正确领导，市人大、市政协监督支持下取得的，凝聚着全市上下、社会各界的智慧和汗水。在此，我代表市人民政府，向所有关心、支持、投身阜新转型、建设、发展的同志们、朋友们，表示衷心的感谢，并致以崇高的敬意！在看到成绩的同时，我们也清醒地看到存在的困难和问题：经济总量小、财政收支矛盾突出的局面还没有根本改变；工业经济占全市经济比重仍然较低，大项目、大企业、大产业集群支撑能力不足；农业现代化水平不高，农村社会事业发展滞后；涉及群众切身利益的问题仍然较多，保障改善民生的任务还很繁重；个别部门和工作人员服务意识有待加强，公务员队伍整体素质需要进一步提高。对这些问题，我们一定要高度重视，认真加以解决。同时，我们诚恳地希望，各位代表、各位委员及时对政府工作提出意见和建议，共同把阜新建设好、发展好。

　　二、“十二五”时期总体要求和目标任务

　　今年，我们开始实施第十二个五年规划，我市将进入跨越发展的新阶段。

　　“十二五”时期的总体要求是：全面贯彻党的十七届五中全会及市委十届十一次全会精神，深入贯彻落实科学发展观，以科学发展、创新发展、和谐发展为主题，以创建全国资源型城市转型示范市为主线，大力实施工业强市、城镇化带动、开放创新、生态立市、文化兴市战略，全力保障和改善民生，维护社会和谐稳定，实现经济社会跨越发展，全面提高建设小康社会水平，让全市人民过上更加美好的幸福生活。

　　奋斗目标是：——转型振兴开创新局面。加快转变经济发展方式，经济转型、社会转型和体制机制转型全面推进，多元化产业格局明显优化，接续替代产业支撑和带动作用明显增强，可持续发展能力明显提升，努力创建全国资源型城市转型示范市。

　　——经济发展实现新跨越。到2015年，地区生产总值达到1000亿元，年均增长20%以上；地方财政一般预算收入突破100 亿元，年均增长27%以上；全社会固定资产投资累计超过5000亿元，年均增长30% 以上。经济总量做大，财政实力做强，主要经济指标增幅高于全省、高于转型以来平均水平。

　　——人民生活达到新水平。覆盖城乡的社会保障体系不断完善，就业稳定性不断提高，历史遗留问题得到较好解决。到2015年，城镇居民家庭人均可支配收入26600 元，年均增长16%；农村居民家庭人均纯收入13500 元，年均增长15.7%。人民生活超过全国平均水平。人民精神文化生活更加丰富，精神文明建设呈现新风貌。

　　为了实现上述目标，我们要深入实施五个战略。

　　——实施工业强市战略，加快推进三次产业协调发展。坚持走新型工业化道路，着力构筑以“三大基地”、“六个重点产业集群”为支撑的多元化产业格局，工业经济比重提高10 个百分点，达到45%，实现由工业化初期向中期的历史性跨越。

　　坚持现代农业发展方向，大力调整农业结构，加快农业产业化进程，扎实推进社会主义新农村建设。坚持把现代服务业作为产业优化升级的重点，以服务业聚集区建设为载体，加快形成区域特色明显、支柱产业突出的发展格局。

　　——实施城镇化带动战略，加快城乡一体化进程。坚持走新型城镇化道路，推进“三位一体”规划深入实施，建设一批重大城乡基础设施工程，促进新城建设与产业发展相融合，完善公共服务配套体系，拓展城镇发展空间。城区实现全域城市化，县城、中心城镇、重点镇建设取得明显成效，城镇化率达到65% 以上，接近全省平均水平，努力建设碧水蓝天、和谐宜居的城市。

　　——实施开放创新战略，加快体制机制转型。以解放思想为先导，以开放增强活力，以改革增强内力，以创新增强动力，以人才释放能量，努力构建充满活力、富有效率、更加开放的体制机制。扩大对外开放，加快形成全方位、宽领域、深层次的对外开放格局。深化各领域改革，努力破除制约经济社会发展的体制性弊端和制度性障碍。加快推进科技创新，科技贡献率达到60%。加强人才队伍建设，创新人才工作机制，以人才优先发展引领经济社会发展。

　　——实施生态立市战略，加快生态文明建设。推进全省生态恢复示范区建设，打造森林城市、绿色村庄，森林覆盖率达到40%。积极发展低碳经济，推行清洁生产，加快发展循环经济。强化节能减排责任制，坚决淘汰落后产能。坚持节约集约利用资源，提高资源综合利用水平，加快建设资源节约型、环境友好型社会。

　　——实施文化兴市战略，加快社会事业发展。传承和挖掘阜新特色文化，弘扬富有时代精神和地域特征、以“创业、创新、创优”为核心的阜新精神，塑造城市文化品牌，繁荣文化事业，发展文化产业，完善文化基础设施和服务体系，将文化的精神力量转化为物质力量。加强社会建设，促进教育、卫生、体育等各项事业快速发展。社会管理体系更加完善，民主法制更加健全，社会更加和谐稳定。

　　“十二五”时期，将是我市实现跨越发展的战略机遇期，是经济、社会、体制机制的全面转型期，是总量提升、缩小差距的发展追赶期。我们要以更开放的理念，更科学的方法，更有力的措施，全力推进经济社会发展实现新跨越。经过“十二五”的努力，全市一定会经济发展得更快，城市建设得更美，人民生活过得更好。

　　三、2011 年工作安排

　　2011 年是“十二五”的开局之年，是“决战500天，实现跨越发展”的决胜之年。做好今年工作，意义重大，影响深远。

　　全市经济社会主要预期目标是：地区生产总值440 亿元，增长20%；地方财政一般预算收入41亿元，增长36.4%；全社会固定资产投资600 亿元，增长71.4%；社会消费品零售总额180 亿元，增长20.9%；直接利用外资1.3 亿美元，增长18.2%；出口总额1.45亿美元，增长20.8%；城镇居民家庭人均可支配收入14720元，增长16%；农村居民家庭人均纯收入7280 元，增长12%；居民消费价格总水平涨幅控制在3%以内。

　　在实际工作中，为了体现跨越发展，上述目标我们不但要必保完成，而且要超额完成，力争地区生产总值达到500 亿元，地方财政一般预算收入达到50亿元，全社会固定资产投资达到700 亿元，全面完成“决战500 天，实现跨越发展”的各项目标任务。

　　（一）做大工业经济总量，加快工业化进程

　　实施工业强市战略，大力推进工业“五项工程”，工业经济占全市经济比重提高2个百分点。规模以上工业增加值达到152 亿元，增长30%。

　　继续开展“千个项目大会战”，实施投资千万元以上项目1200个，其中新开工600个、续建600个。全年竣工投产项目400个。

　　“ 三大基地”建设要有重大进展。大唐煤制气一期项目完成投资100亿元，60亿立方米煤制气二期等项目开展前期工作，打造煤化工示范基地。力争巴新铁路试通车及复线工程核准。新开工风电项目70 万千瓦，风电装机容量突破200 万千瓦。开工建设兆瓦级光伏发电示范项目，启动大金重工风机总装及新疆金风风电设备制造产业园等项目。开工建设辉山乳业集群等项目，推进雨润畜禽产品产业园等项目竣工投产。

　　“六个重点产业集群”建设要实现新突破，新开工项目385个，产值均实现50%以上增长，总产值达到400亿元以上。

　　实施企业“提升工程”、“倍增计划”和新上项目“达产达效行动”，新增规模以上工业企业100 户、主营业务收入超亿元企业20 户。阜矿集团总收入突破200 亿元，开工建设彰武雷家煤田，推进膨润土深加工等项目竣工投产。

　　拓宽民营经济发展领域，完善政策支持和社会化服务体系，着力提高企业竞争力，做强民营骨干企业，支持中小企业加快发展，推进全民创业，民营经济增加值、税金均增长30%。

　　（二）以新城建设为重点，加快城镇化进程

　　实施城镇化带动战略，以“三位一体”规划为载体，以城区全域城市化和农民工市民化为突破口，以城乡基本公共服务优化配置为依托，提高城镇化发展水平。

　　新城建设要呈现新形象。“玉龙新城”施工面积达到114万平方米，加快道路、水系、管网等基础设施和市民回迁楼等项目建设，推进规划展示馆、博物馆、市民服务中心等项目建设。启动城市南部综合体项目。开工建设“沈彰新城”“三横四纵”道路及柳河治理等工程，沈彰开发大道竣工通车。加快“温泉旅游新城”、“现代皮革新城”、“蒙古贞新城”基础设施和重点项目建设步伐。搞好县、镇、村城镇化试点工作，提高县城和小城镇环境承载力、要素集聚能力。做大做强建筑业，产值突破百亿元。

　　加快阜盘高速、彰通高速建设步伐，推进京沈客专、锦阜高铁路扩能改造、阜盘高速北延伸线等工程开工，军民合用机场、沈彰通客专等项目前期工作要取得积极进展。继续实施道路畅通工程，皮革基地快速通道竣工通车，建设新邱开发大道、阜蒙县客运站，改造新建中华路东段、西山路、滨湖路等30 条街路。更新公交车辆66 台，新增、改造公交线路7 条。实施供水、供热等管网改造工程。

　　加大城市绿化力度，新增城市绿化面积400万平方米。加快环城水系建设，实施细河改造，搞好玉龙河等工程建设，新增水面22.4万平方米。以创建全国卫生城为目标，开展重点部位专项整治和义务清扫活动，提升城市环境质量。扩大小区物业覆盖面，开展示范街路、示范小区创建活动，不断提高城市管理水平。

　　（三）大力发展县域经济，加快现代农业发展步伐

　　实施新一轮县域经济“三年倍增计划”，大力推进县域工业化、城镇化、农业现代化进程，积极培育县域主导产业和特色产业，做大县域经济总量。坚持“生产发展、生活宽裕、乡风文明、村容整洁、管理民主”方针，推进社会主义新农村建设。

　　大力调整农业结构，加快农产品加工企业配套基地建设步伐。新建保护地5万亩，力争10万亩；花生种植面积达到250万亩。着力发展生猪、奶牛、肉牛、肉羊、肉禽等重点农产品基地，新建畜禽标准化养殖小区300 个。培育壮大甜玉米、马铃薯、南瓜、树莓、酿酒葡萄等特色农产品基地。加强农产品质量安全监管和无规定动物疫病示范区建设。

　　新建及改善灌溉面积9.5万亩，新增流域生态综合治理面积20 万亩，继续搞好病险水库除险加固工程。增强气象服务“三农”的能力。

　　实施机械化保护性耕作130万亩。

　　（四）壮大城区经济规模，提升服务业发展水平

　　以服务业聚集区建设为重点，积极培育新的经济增长点，完成城区经济“三年倍增计划”。

　　推进中央商务区建设，东市地下商业街、解放大街地下商业城投入使用，开工建设兴隆百货二期等一批项目，促进一批知名品牌尽快落户。加快站前和迎宾大街商务聚集区建设，集中打造一批专业市场和特色商业街区。对花园市场进行改造升级，建设蔬菜批发市场三期工程。继续实施“万村千乡”市场工程、“家电下乡”、“家电以旧换新”等惠民工程。

　　积极发展房地产、现代物流、电子商务、文化旅游等新兴服务业。

　　房地产施工面积500 万平方米，增长25%。加快国际物流园建设，开工建设葫芦岛港物流产业园区，启动营口内陆港、交通物流园和煤炭物流基地等项目。进一步做大旅游业，启动葛根苏木旅游度假区项目、海州露天国家矿山公园晋升国家4A级景区工作，加大孙家湾国际工业遗产旅游度假区、海棠山、瑞应寺、大清沟等景区开发力度，建设金石温泉康复疗养中心、奇石博物馆等项目。积极发展民族风情游和乡村游。

　　扩大消费需求，提高收入水平，促进消费能力升级，活跃农村消费市场。采取综合性政策措施，加强价格监管，抓好“菜篮子”，管好“米袋子”，保障市场供应，维护物价基本稳定。

　　（五）加大招商引资力度，全面扩大对外开放

　　实施开放创新战略，推进项目、资金、技术、先进理念的全方位引进，促进全面开发开放。

　　坚持“走出去”与“请进来”相结合，突出主题概念和产业集群招商，组织好重大招商活动。加强与大企业、大集团对接合作，积极引进重大项目和知名品牌。发挥县区专业化招商、部门抓产业和企业招商主体作用，提高招商成效。强化重大项目包保责任制，加快项目落地、开工进程。开发储备一批打基础、利长远、带动能力强的战略项目。全年招商引资到位额230亿元，增长53%。

　　拓宽利用外资渠道，加大装备制造业、新能源、现代服务业等领域的外资引入力度。优化出口产品结构，做大外贸骨干企业，培育新的出口增长点。加快沈阜产业带规划实施，加强与沈阳经济区其他城市在基础设施、经贸、旅游、融资等领域的合作。

　　（六）做好财税金融工作，为经济社会发展提供资金保障

　　强化税收征管，做到依法征收、应收尽收。继续加强对采矿、建筑、房地产、餐饮娱乐等重点行业和领域的税收征管，严厉打击各种偷税、逃税、抗税等违法行为。加强非税收入征管，提高政府统筹能力。基本完成各类国有中小企业的产权制度改革，提高国有资产和资源经营管理水平。严格控制一般性支出，降低行政成本。加强对政府投资类项目的财政预算审查，强化财政和审计监督。全力向上争取政策、资金、项目支持，全面落实省直部门、银行帮扶工作。

　　加强金融环境建设，推进多渠道融资，贷款融资额突破200 亿元。新建阜新银行大连分行，引进交通银行等金融机构。利用好股权投资基金、信托、租赁等融资渠道，加快德尔、振隆、华通等企业上市进程。小额贷款公司累计投放突破20 亿元，担保机构达到10家以上。加强融资平台建设，提高各级政府融资能力。启动城市“一卡通”工程。

　　支持保险业快速发展。

　　（七）加强环境保护和资源节约，提高生态文明建设水平

　　实施生态立市战略，促进经济发展方式转变，增强可持续发展能力。

　　全面启动生态恢复示范区建设，实施人工造林和封山育林100 万亩，加快打造森林城市、绿色村庄。巩固和扩大封山禁牧、“小开荒”治理成果，全面开展国有林场改革。

　　大力发展循环经济，加快再生资源循环经济产业基地建设，入驻项目50个。提高粉煤灰、煤矸石综合利用率，抓好阜新发电公司粉煤灰综合利用等一批重点项目。严格落实节能减排责任制，推进重点领域节能降耗，促进高耗能企业改造升级，加快淘汰落后产能。

　　坚持节约集约用地，提高土地利用率和投资强度、产出效益，加大闲置土地和低效利用土地清理力度，切实用好土地增减挂钩政策，合理有序开发土地和矿产资源。加强海州露天矿、清河门矿、东梁矿环境治理。强化城市烟尘整治，加大对污染源的取缔和改造力度，提高城市空气质量。改善水环境，促进水资源优化配置。

　　（八）抓好重点民生工程，让发展成果更好地惠及民众

　　政府的一切工作都是为了让老百姓过上好日子，让全市人民在推动转型发展的过程中感受快乐、和谐和幸福。

　　多渠道开发就业岗位，以创业带动就业，重点抓好就业困难群体就业援助，促进高校毕业生充分就业，全年实名制就业5万人，加快省级创业型城市建设。推动企业开展工资集体协商工作。切实保障失地农民合法权益。

　　继续扩大社会保险覆盖面，加强基金征缴管理工作。提高城镇职工养老保险待遇标准，扩大城镇居民基本医疗保险覆盖面，进一步解决困难企业退休人员医疗保险问题，继续推进新型农村社会养老保险试点工作。提高城乡居民最低生活保障水平和新农合报销比例、资金使用率。

　　完善社区“ 十分钟服务圈”功能，组织好第八届社区居委会换届选举工作。积极开展农村示范社区建设。继续推进社会福利、慈善事业发展，启动市社会养老院、老年活动中心、社会福利中心、农村常年病人托管中心等项目。

　　全年办好与人民群众生活密切相关的15 件实事。（1）维修改造住宅小区与城区主干路连接路50 条。（2）全部解决城市中心区无灯路问题。（3）在城市中心区新建公厕46 座。（4）新建、改造市民健康广场50 个，三•一八公园免费向市民开放。（5）实施老旧住宅小区综合整治169 万平方米，三年整治工程全面完成。（6）实施居民楼供热计量和节能改造80 万平方米。（7）以清河门矿区、东梁矿区为重点，建设棚户区改造回迁楼253万平方米，其中，建设廉租住房、经济适用住房25万平方米。（8）新建和续建农村饮水安全工程220处，解决10万人饮水安全问题。（9）修建中华路地下人行通道。（10）新增养老床位500 张。（11）新建1.5万座农村无害化厕所。（12）对40 个贫困村实施整村推进扶贫。

（13）新建农村乡镇综合文化站28 个，实现乡镇全覆盖。（14）新建、改造农村学校塑胶操场4 个。（15）改造维修城乡校舍5万平方米。这些涉及民生的实事，我们都要快办、办好，说到做到，不打折扣，全部兑现。

　　（九）统筹经济社会协调发展，加强社会建设

　　实施文化兴市战略，以文化软实力激发全市人民的凝聚力和创造力。开展“创业、创新、创优”活动，大力培育先进文化。深入推进文化惠民工程，活跃城乡文化生活。加快公共文化设施和服务体系建设，市图书馆投入使用，建设阜新传媒大厦，启动群众文化中心项目。积极培育文化骨干企业，繁荣文化市场。开展做文明市民活动，进一步提高市民综合素质。

　　树立人才强市理念，加大人才工作力度，以高层次人才、高技能人才为重点，统筹推进各类人才队伍建设。创新人才培养、使用、评价、激励机制，搭建聚才、育才、创业平台，积极营造有利于人才脱颖而出的制度环境。

　　全面推进重点产业集群创新服务体系建设，打造科技创新服务平台。加快高新技术企业培育，努力提高企业自主创新能力。推进与辽工大全面合作，加快液压研发检测平台建设。高新技术产品增加值达到18亿元。

　　深入推进新一轮“普九”工作，启动中小学教育信息化“班班通”工程，促进义务教育均衡发展。积极推进普通高中优质特色发展。大力发展职业教育，加强专业技能人才培养，改善教育培训条件，力争二职专新校区投入使用。加快发展学前教育，鼓励规范民办教育。进一步深化课堂教学改革，提高教育教学质量。加强师德师风建设，提升教师队伍素质。

　　推进医药卫生体制改革，完善城乡卫生监督、疾病预防控制体系和医疗救治体系。开工建设阜矿总医院内科大楼和第四、第五人民医院病房楼。做好人口和计划生育工作，不断提高出生人口素质。

　　广泛开展群众性体育活动，促进群众体育、竞技体育和体育产业全面发展，加快建设全国一流篮球城。做好承办第十二届全运会部分赛事筹备工作。

　　支持蒙医、蒙药等民族产业发展，改善民族教育办学条件，继续办好敖包文化节，推动民族事业进步。充分发挥宗教界人士和信教群众在促进社会和谐中的积极作用。

　　自觉接受人大依法监督和政协民主监督，认真办理人大代表建议和政协提案，认真听取各民主党派、工商联、无党派人士和各人民团体的意见，发挥好工会、共青团、妇联桥梁纽带作用，主动接受社会公众和新闻舆论监督。增强依法行政意识，提高依法行政能力。

　　加强社会治安综合治理，遏制各类违法犯罪活动。完善应急管理体系，科学有效应对和处置各类突发事件。畅通信访渠道，及时化解社会矛盾。全面落实安全生产责任制，杜绝重特大事故发生。加强对食品药品源头监控和流通监管，保障食品药品安全。做好“六五”普法工作。开展好双拥模范城创建活动。

　　（十）加强政府自身建设，打造人民满意的服务型政府

　　“决战500 天，实现跨越发展”对各级政府和全体公务员提出了超常要求。我们必须采取超常的举措，付出超常的努力，切实提高施政水平，更好地为“突破阜新”大局、为全市人民服务，努力打造人民满意的服务型政府。

　　打造人民满意的服务型政府，必须坚持解放思想，勇于创新。以时不我待的紧迫感、慢进则退的危机感、拼搏争先的责任感，奋发有为，开拓进取。各级干部要加强学习，将学习作为提升素质、增强能力的第一需要，作为考核、衡量干部工作的重要内容。各项工作要保持连续性，突出创造性，体现先进性。要思想上加压、工作上加力、发展上加速。

　　打造人民满意的服务型政府，必须坚持狠抓落实，务求实效。要做到重落实，敢落实，善落实。提倡现场工作法，深入一线，靠前指挥，在抓落实中发现问题，解决矛盾，创造业绩。进一步深化政府绩效考评机制，把工作任务细化、量化、责任化，使政府各项工作分工更加明确，责任更加强化，执行更加有效。

　　打造人民满意的服务型政府，必须坚持效能优先，雷厉风行。要发扬只争朝夕精神，各项工作尽可能往前抢、争主动，以高效率推进工作开展。要进一步精简会议，大力改善文风会风，坚持说短话、开短会、发短文。各部门的工作都要体现满负荷、快节奏、高强度。

　　打造人民满意的服务型政府，必须坚持大局为重，合力攻坚。要强化“一盘棋”思想，在全市唱响跨越发展主旋律和最强音。要调动一切积极因素，凝聚各方智慧和力量，全市上下主动配合，相互支持，同心协力，共同干事创业。各级各部门都要集中精力，把职责范围内的事情办好，把应该办、能够办、可以办的事情办好，做到矛盾不上交、困难不回避、责任不推诿，提升政府执行力。

　　打造人民满意的服务型政府，必须坚持廉洁从政，干净干事。一个有权威、得民心的政府，一定是干净廉洁的政府。各级领导干部要按照“为民、务实、清廉”的要求，切实做到有作为、有操守，既干事、又干净，常怀敬畏之心、常思贪欲之害、常修为政之德。要加强监督检查，充分发挥监察等部门的作用。严格落实廉政建设责任制，深入开展专项治理，严肃查处违法违纪案件，以廉洁的形象取信于民。

　　各位代表！ 突破阜新正当其时，跨越发展势不可挡！让我们在市委的正确领导下，以敢于攻坚的坚强决心，以战则必胜的坚定信心，以勇攀高峰的壮志雄心，凝心聚力，乘势而上，建设好我们共同的美好家园，为实现阜新跨越发展的宏伟目标而努力奋斗！
